# Constructing a fall risk prediction model for hospitalized patients using machine learning

**DOI:** 10.1186/s12889-025-21284-8

**Published:** 2025-01-20

**Authors:** Cheng-Wei Kang, Zhao-Kui Yan, Jia-Liang Tian, Xiao-Bing Pu, Li-Xue Wu

**Affiliations:** 1 Department of Orthopaedics, West China School of Public Health and West China Fourth Hospital, Sichuan University, Chengdu, Sichuan 610041 China; 2 Department of Pathology, West China School of Public Health and West China Fourth Hospital, Sichuan University, Chengdu, Sichuan 610041 China

**Keywords:** Accidental falls, Hospitalized patients, Risk factors, Machine learning, Predictive modeling, Model interpretation

## Abstract

**Study objectives:**

This study aimed to identify the risk factors associated with falls in hospitalized patients, develop a predictive risk model using machine learning algorithms, and evaluate the validity of the model’s predictions.

**Study design:**

A cross-sectional design was employed using data from the DRYAD public database.

**Research methods:**

The study utilized data from the Fukushima Medical University Hospital Cohort Study, obtained from the DRYAD public database. 20% of the dataset was allocated as an independent test set, while the remaining 80% was utilized for training and validation. To address data imbalance in binary variables, the Synthetic Minority Oversampling Technique combined with Edited Nearest Neighbors (SMOTE-ENN) was applied. Univariate analysis and least absolute shrinkage and selection operator (LASSO) regression were used to analyze and screen variables. Predictive models were constructed by integrating key clinical features, and eight machine learning algorithms were evaluated to identify the most effective model. Additionally, SHAP (Shapley Additive Explanations) was used to interpret the predictive models and rank the importance of risk factors.

**Results:**

The final model included the following variables: Adl_standing, Adl_evacuation, Age_group, Planned_surgery, Wheelchair, History_of_falls, Hypnotic_drugs, Psychotropic_drugs, and Remote_caring_system. Among the evaluated models, the Random Forest algorithm demonstrated superior performance, achieving an AUC of 0.814 (95% CI: 0.802–0.827) in the training set, 0.781 (95% CI: 0.740–0.821) in the validation set, and 0.795 (95% CI: 0.770–0.820) in the test set.

**Conclusion:**

Machine learning algorithms, particularly Random Forest, are effective in predicting fall risk among hospitalized patients. These findings can significantly enhance fall prevention strategies within healthcare settings.

**Supplementary Information:**

The online version contains supplementary material available at 10.1186/s12889-025-21284-8.

## Introduction

Due to illness [1], medication [2], unfamiliar environments [3], and physical limitations [4], and other factors, hospitalized patients are particularly vulnerable to falls. Falls can lead to serious injury, prolonged hospitalization, functional decline, increased healthcare costs, disability, and even death. The incidence of falls among hospitalized patients is reported to be three times higher than that of community-dwelling residents aged 65 years and older [5]. In the U.S. alone, falls among older adults result in tens of billions of dollars in increased healthcare costs each year [6]. Recognizing the harmful consequences of falls, healthcare providers are increasingly focused on implementing fall prevention programs. However, despite these efforts, falls remain a significant challenge for healthcare organizations.

Understanding the risk factors for falls in hospitalized patients is critical for healthcare providers to identify high-risk populations and implement targeted prevention strategies. Preventing falls and injuries is challenging due to the complexity of falls and injuries, as well as the combination of patients’ underlying diseases and disabilities. Traditional fall risk assessment methods such as the Morse Falls Scale (MFS) [7], may not fully capture all risk factors associated with falls in hospitalized patients. Whereas the use of medications such as psychotropic medications, antihypertensive medications, and the use of tricyclic antidepressants have been identified as risk factors for falls in hospitals [8, 9].

Previous studies have used machine learning algorithms to improve the accuracy of fall risk prediction. For example, Palumbo et al. utilized support vector machines and decision trees to predict fall risk in older adults, achieving a high degree of accuracy [10]. Choi JH et al. used a machine learning algorithm with AUROC values that were similar to the MFS in predicting the risk of falls in patients with acute stroke, thus allowing for accurate and effective fall screening [11]. These studies underscore the potential of machine learning in fall risk prediction but also highlight the need for further research to refine these models and improve their generalizability.

Our study contributes to this growing body of literature by developing a comprehensive predictive model that integrates multiple machine learning algorithms, including Logistic Regression, Random Forest, and XGBoost. Unlike previous studies, our model incorporates a wider range of clinical and demographic variables, including activities of daily living (ADLs), cognitive dysfunction, and medication use, which have been shown to significantly impact fall risk. Additionally, we employ SHAP (SHapley Additive ExPlanations) to provide a more interpretable model, allowing clinicians to better understand the contribution of each variable to fall risk. This approach not only enhances the predictive power of our model but also provides actionable insights for healthcare providers.

This study aims to construct a predictive model of fall risk in hospitalized patients using machine learning algorithms. Early identification of these risk factors, focusing on patients using specific medications, and early interventions to prevent falls may be effective. This will inform clinicians in the early identification of high-risk patients at the time of admission and provide effective recommendations for public health policymakers in further policy development.

## Methods and materials

The data used in this study were obtained from the open-access DRYAD database, specifically the dataset titled “Risk factors of falls in inpatients…” (10.1136/bmjopen-2014-005385). All private information was anonymized, and the data collection process complied with the Declaration of Helsinki. Ethical approval was obtained from the Ethics Committee of The West China Fourth Hospital of Sichuan University. According to Article 32 of the Measures for Ethical Review of Life Sciences and Medical Research Involving Humans (Document No. Guo Wei Ke Jiao Fa [2023] 4), the research qualifies as [briefly describe type of research, e.g., “analysis of anonymized data” or “use of publicly available data”]. As it does not involve direct interaction with individuals or the use of private or sensitive information, informed consent was not required.

### Methodology

This study employed a cross-sectional design and conducted a secondary analysis using data from the Fukushima Medical University Hospital (FMUH) database in Japan.

### Research variables and definitions

The dataset utilized in this study comprises a comprehensive set of 29 clinical variables that are potentially associated with falls in hospitalized patients. These variables include sex, age, fall event, days of occurrence, planned surgery, history of falls, assistance with activities of daily living (ADLs), wheelchair use, mobility assistance, rehabilitation, impaired manual muscle test (MMT), laxative, sedative, psychotropic and hypnotic medication use, remote caring system, cognitive dysfunction, eyesight, bed sensor, age group, inhibition, and days spent in the hospital.

To ensure the robustness of the analysis, certain variables were excluded. The variable “days of occurrence” associated with fall events was excluded because hospitalization days may significantly prolong after a fall. Additionally, the variable “days in hospital” was removed. As a result, a total of 27 variables were included in the analysis, and after excluding the dependent variable of fall event, there remained 26 predictors.

The ADL variable is categorized into five levels: 0 = Independent, 1 = Partially Dependent, 2 = Moderately Dependent, 3 = Severely Dependent, and 4 = Totally Dependent. This classification system helps assess the patient’s level of dependency in performing daily activities. The impaired manual muscle test is classified into six grades, ranging from lowest to highest: 0 = Zero, 1 = First (Poor), 2 = Second (Fair), 3 = Third (Good), 4 = Fourth (Normal), and 5 = Fifth (Excellent). This grading system is used to evaluate the patient’s muscle strength and function.

### Multicollinearity analysis

To evaluate potential multicollinearity among the predictor variables, variance inflation factor (VIF), conditional index (CI), and variance decomposition proportion (VDP) were calculated. All analyses were conducted to ensure that the included variables did not show problematic levels of multicollinearity, which could bias model performance.

### Construction and evaluation of predictive models

In this study, patients were categorized into two groups based on the occurrence of fall events: a non-fall group and a fall group. To ensure the robustness of the analysis, 80% of the dataset was used for training and validation, while the remaining 20% was allocated as an independent test set. Statistical comparisons between the two groups were performed to identify variables with significant differences. To address the issue of data imbalance in the training set, we applied the Synthetic Minority Oversampling Technique combined with Edited Nearest Neighbors (SMOTE-ENN). This method effectively mitigated the imbalance and improved the model’s robustness. Detailed benchmark data for the training and test sets are provided in Tables S1 and S2, respectively, while Table S3 presents the distribution of the non-fall and fall groups in the training set after SMOTE-ENN treatment.

To identify the most relevant predictors, exploratory data analyses were conducted. First, heatmaps were employed to examine variable correlations, and a correlation matrix (CM) was used to check for multicollinearity issues among the features. Subsequently, univariate analysis was performed using the chi-square test for categorical variables (e.g., wheelchair use) and the Mann-Whitney U test for continuous variables (e.g., age) to assess significant differences between the fall and non-fall groups. Refer to Supplementary Table S4.

For further feature selection, we employed a feature selection algorithm. Specifically, we utilized Least Absolute Shrinkage and Selection Operator (LASSO) regression with a binomial distribution to accommodate the binary nature of fall events (The variables selected by LASSO and the hyperparameter tuning details are presented in Supplementary Table S5). To comprehensively assess the predictive performance of the selected features, eight machine learning classification models were employed: Extreme Gradient Boosting (XGBoost), Logistic Regression (LR), Random Forest (RF), Gradient Boosted Decision Tree (GBDT), Gaussian Naive Bayes (GNB), Multi-Layer Perceptron (MLP), Support Vector Machines (SVM), and K-Nearest Neighbors (KNN). Model performance and predictive accuracy were evaluated using various metrics, including area under the receiver operating characteristic curve (AUC), accuracy, sensitivity, specificity, positive predictive value (PPV), negative predictive value (NPV), F1 score, and Cohen’s Kappa. AUC, being one of the most critical metrics [12], was employed to gauge the overall model performance.

**Fig. 1:**
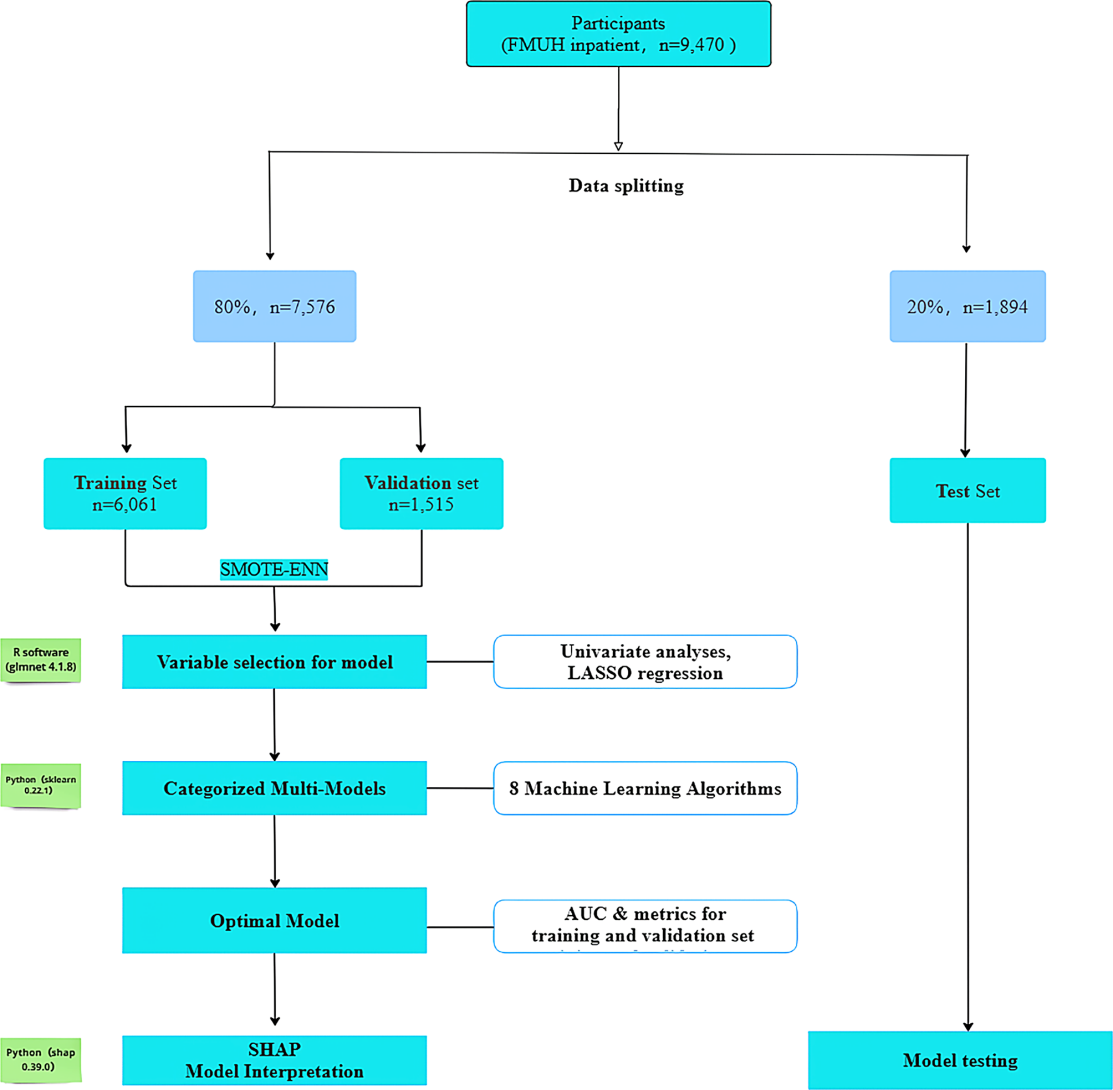
Flowchart of predictive model construction

Furthermore, we implemented the SHapley Additive exPlanations (SHAP) method to interpret the predictive outcomes of the machine learning models. SHAP quantifies the contribution of each feature to the prediction outcomes and ranks the importance of the predictors. Figure 1 presents a flowchart outlining the methodological process.

### Statistical analysis and software packages

Categorical variables were presented as counts and percentages, and comparisons between groups were conducted using the chi-square test. For continuous variables, we first performed a normality test (Shapiro-Wilk test) to determine if the data followed a normal distribution. Based on the results of the normality test, we used the t-test for normally distributed variables and the Mann-Whitney U test for variables that did not follow a normal distribution. Continuous variables were expressed as mean ± standard deviation (SD) for normally distributed data, and median with interquartile range (IQR) for non-normally distributed data. A two-tailed test was used to assess the level of statistical significance, with *P* < 0.05 considered significant. The statistical analyses and corresponding software packages are detailed in Table 1.

**Table 1:** Main statistical methods and associated software packages used in this study

Analysis/Model	Software/Language	Package
Statistical Analyses	SPSS (version 22.0)	IBM Corp., Armonk, NY, USA
LASSO regression	R (version 4.2.3)	glmnet (version 4.1.8)
SHAP Model	Python (version 3.7)	shap (version 0.43.0)
Random Forest (RF)	Python (version 3.7)	scikit-learn (version 0.20.1)
SMOTE-ENN Balancing	Python (version 3.7)	imbalanced-learn

## Results

### Comparison of baseline data

The dataset consisted of a total of 9,470 subjects, including 4,748 males and 4,722 females. We divided the subjects into two groups based on the occurrence of a fall event during hospitalization. Among the subjects, 9,240 experienced no falls, while 230 had falls, resulting in a fall incidence rate of 2.43%.

The median age (interquartile range) of individuals who experienced falls was 72 years (range, 61–78 years). The median number of days (interquartile range) until a fall occurred was 14 days (range, 5–27 days). Additionally, the median length of hospitalization (interquartile range) for the falls population was 25 days (range, 10–45 days), which was significantly longer compared to the median hospitalization time of 10 days (range, 4–19 days) for the population without falls.

A detailed summary of patient demographics and the statistical differences between the two groups are presented in Table 2.

**Table 2:** Summary of baseline data analysis results

Variable	Group	Totals (n = 9470)	Fallevent = NO (n = 9240)	Fallevent = YES (n = 230)	Statistic	*P*-value^2^
Sex, n (%)	Male	4748 (50.137)	4638 (50.195)	110 (47.826)	0.504	0.478
	Female	4722 (49.863)	4602 (49.805)	120 (52.174)		
Planned surgery, n (%)	No	5475 (58.381)	5311 (58.025)	164 (72.889)	19.969	< 0.001
	YES	3903 (41.619)	3842 (41.975)	61 (27.111)		
Fall history, n (%)	NO	8631 (91.246)	8461 (91.678)	170 (73.913)	88.672	< 0.001
	YES	828 (8.754)	768 (8.322)	60 (26.087)		
ADL_standing, n (%)	0	7987 (84.367)	7852 (85.006)	135 (58.696)	132.755	< 0.001
	1	264 (2.789)	243 (2.631)	21 (9.130)		
	2	257 (2.715)	235 (2.544)	22 (9.565)		
	3	210 (2.218)	195 (2.111)	15 (6.522)		
	4	749 (7.912)	712 (7.708)	37 (16.087)		
ADL_sitting, n (%)	0	8377 (88.505)	8205 (88.847)	172 (74.783)	54.797	< 0.001
	1	112 (1.183)	107 (1.159)	5 (2.174)		
	2	194 (2.050)	178 (1.927)	16 (6.957)		
	3	209 (2.208)	196 (2.122)	13 (5.652)		
	4	573 (6.054)	549 (5.945)	24 (10.435)		
ADL_dressing, n (%)	0	7704 (81.377)	7577 (82.029)	127 (55.217)	108.580	< 0.001
	1	96 (1.014)	90 (0.974)	6 (2.609)		
	2	759 (8.017)	711 (7.697)	48 (20.870)		
	3	376 (3.972)	354 (3.832)	22 (9.565)		
	4	532 (5.620)	505 (5.467)	27 (11.739)		
ADL_eating, n (%)	0	8096 (85.518)	7939 (85.948)	157 (68.261)	74.379	< 0.001
	1	87 (0.919)	77 (0.834)	10 (4.348)		
	2	345 (3.644)	324 (3.508)	21 (9.130)		
	3	154 (1.627)	147 (1.591)	7 (3.043)		
	4	785 (8.292)	750 (8.120)	35 (15.217)		
ADL_toileting, n (%)	0	7675 (81.097)	7553 (81.796)	122 (53.043)	134.426	< 0.001
	1	406 (4.290)	383 (4.148)	23 (10.000)		
	2	352 (3.719)	330 (3.574)	22 (9.565)		
	3	413 (4.364)	379 (4.104)	34 (14.783)		
	4	618 (6.530)	589 (6.379)	29 (12.609)		
ADL_evacuation, n (%)	0	7726 (81.610)	7604 (82.321)	122 (53.043)	139.147	< 0.001
	1	302 (3.190)	284 (3.075)	18 (7.826)		
	2	407 (4.299)	382 (4.136)	25 (10.870)		
	3	490 (5.176)	451 (4.883)	39 (16.957)		
	4	542 (5.725)	516 (5.586)	26 (11.304)		
ADL_washface, n (%)	0	7828 (82.696)	7696 (83.326)	132 (57.391)	112.867	< 0.001
	1	210 (2.218)	199 (2.155)	11 (4.783)		
	2	606 (6.402)	562 (6.085)	44 (19.130)		
	3	346 (3.655)	326 (3.530)	20 (8.696)		
	4	476 (5.029)	453 (4.905)	23 (10.000)		
Wheelchair, n (%)	No	7601 (80.272)	7483 (80.994)	118 (51.304)	124.913	< 0.001
	YES	1868 (19.728)	1756 (19.006)	112 (48.696)		
Needs help to move, n (%)	No	8127 (85.827)	7992 (86.503)	135 (58.696)	142.656	< 0.001
	YES	1342 (14.173)	1247 (13.497)	95 (41.304)		
Rehabilitation, n (%)	No	9216 (97.369)	8999 (97.445)	217 (94.348)	8.401	0.004
	YES	249 (2.631)	236 (2.555)	13 (5.652)		
MMT_Right, n (%)	0	83 (0.882)	80 (0.871)	3 (1.304)	58.751	< 0.001
	1	75 (0.797)	69 (0.751)	6 (2.609)		
	2	65 (0.690)	61 (0.664)	4 (1.739)		
	3	101 (1.073)	98 (1.067)	3 (1.304)		
	4	453 (4.811)	421 (4.584)	32 (13.913)		
	5	8638 (91.747)	8456 (92.063)	182 (79.130)		
MMT_Left, n (%)	0	82 (0.870)	81 (0.881)	1 (0.437)	60.171	< 0.001
	1	86 (0.913)	80 (0.870)	6 (2.620)		
	2	74 (0.785)	70 (0.762)	4 (1.747)		
	3	123 (1.306)	119 (1.295)	4 (1.747)		
	4	431 (4.575)	399 (4.341)	32 (13.974)		
	5	8625 (91.551)	8443 (91.852)	182 (79.476)		
Laxative, n (%)	NO	7800 (82.418)	7633 (82.662)	167 (72.609)	15.651	< 0.001
	YES	1664 (17.582)	1601 (17.338)	63 (27.391)		
Remote caring system, n (%)	NO	9391 (99.208)	9170 (99.285)	221 (96.087)	29.207	< 0.001
	YES	75 (0.792)	66 (0.715)	9 (3.913)		
Cognitive dysfunction, n (%)	NO	9231 (97.497)	9022 (97.662)	209 (90.870)	42.423	< 0.001
	YES	237 (2.503)	216 (2.338)	21 (9.130)		
Eyesight group, n (%)	Normal	4956 (52.400)	4846 (52.508)	110 (48.035)	13.233	0.004
	Mild	3720 (39.332)	3616 (39.181)	104 (45.415)		
	Moderate	488 (5.160)	485 (5.255)	3 (1.310)		
	Severe	294 (3.108)	282 (3.056)	12 (5.240)		
Sedative, n (%)	NO	9270 (97.888)	9047 (97.911)	223 (96.957)	0.989	0.320
	YES	200 (2.112)	193 (2.089)	7 (3.043)		
Hypnotic, n (%)	NO	8003 (84.509)	7837 (84.816)	166 (72.174)	27.397	< 0.001
	YES	1467 (15.491)	1403 (15.184)	64 (27.826)		
Psychotropic, n (%)	NO	8863 (93.600)	8676 (93.906)	187 (81.304)	59.495	< 0.001
	YES	606 (6.400)	563 (6.094)	43 (18.696)		
Censor of bed, n (%)	YES	9391 (99.208)	9170 (99.285)	221 (96.087)	29.207	< 0.001
	YES	75 (0.792)	66 (0.715)	9 (3.913)		
Rihabilitation, n (%)	NO	9216 (97.369)	8999 (97.445)	217 (94.348)	8.401	0.004
	YES	249 (2.631)	236 (2.555)	13 (5.652)		
Age group, n (%)	≤ 49	2501 (26.413)	2470 (26.734)	31 (13.478)	64.680	< 0.001
	50 ~ 64	2538 (26.803)	2502 (27.081)	36 (15.652)		
	65 ~ 74	2440 (25.768)	2366 (25.609)	74 (32.174)		
	≥ 75	1990 (21.016)	1901 (20.576)	89 (38.696)		
Inhibition, n (%)	NO	9267 (97.856)	9046 (97.900)	221 (96.087)	3.518	0.061
	YES	203 (2.144)	194 (2.100)	9 (3.913)		
Age, median[IQR]	NA	63.000 [48.000, 73.000]	62.000 [48.000, 73.000]	72.000 [61.000, 78.000]	-7.800	< 0.001
Days of occurrence. median[IQR]	NA	0.000[0.000,0.000]	0.000[0.000,0.000]	14.000 [5.000,27.000]	-25.946	< 0.001
Days in hospital. median[IQR]	NA	10.000 [4.000,19.000]	10.000 [4.000,19.000]	25.000 [10.000, 45.000]	-10.003	< 0.001

The median (interquartile range, IQR) or frequency (%) was calculated. The Wilcoxon rank sum test, Pearson’s chi-squared test, and Fisher’s exact test were used for the analysis. For variables such as age, days of occurrence, and days in the hospital, the Mann-Whitney U test was employed. The chi-square test was utilized for the remaining variables

### Multicollinearity results

The variance inflation factor (VIF) values for all predictor variables were below 5, indicating no significant multicollinearity. Specifically, variables such as Adl_standing and Adl_evacuation had slightly higher VIF values compared to others but were still within the acceptable range. The conditional index (CI) values were all below 10, with the highest value being 2.80, suggesting minimal covariance. Additionally, the variance decomposition proportion (VDP) analysis showed no variable with consistently high values, supporting the inclusion of these variables in the predictive model.

### Screening for factors indicating fall risk

#### Exploratory data analysis (EDA)

**Fig. 2:**
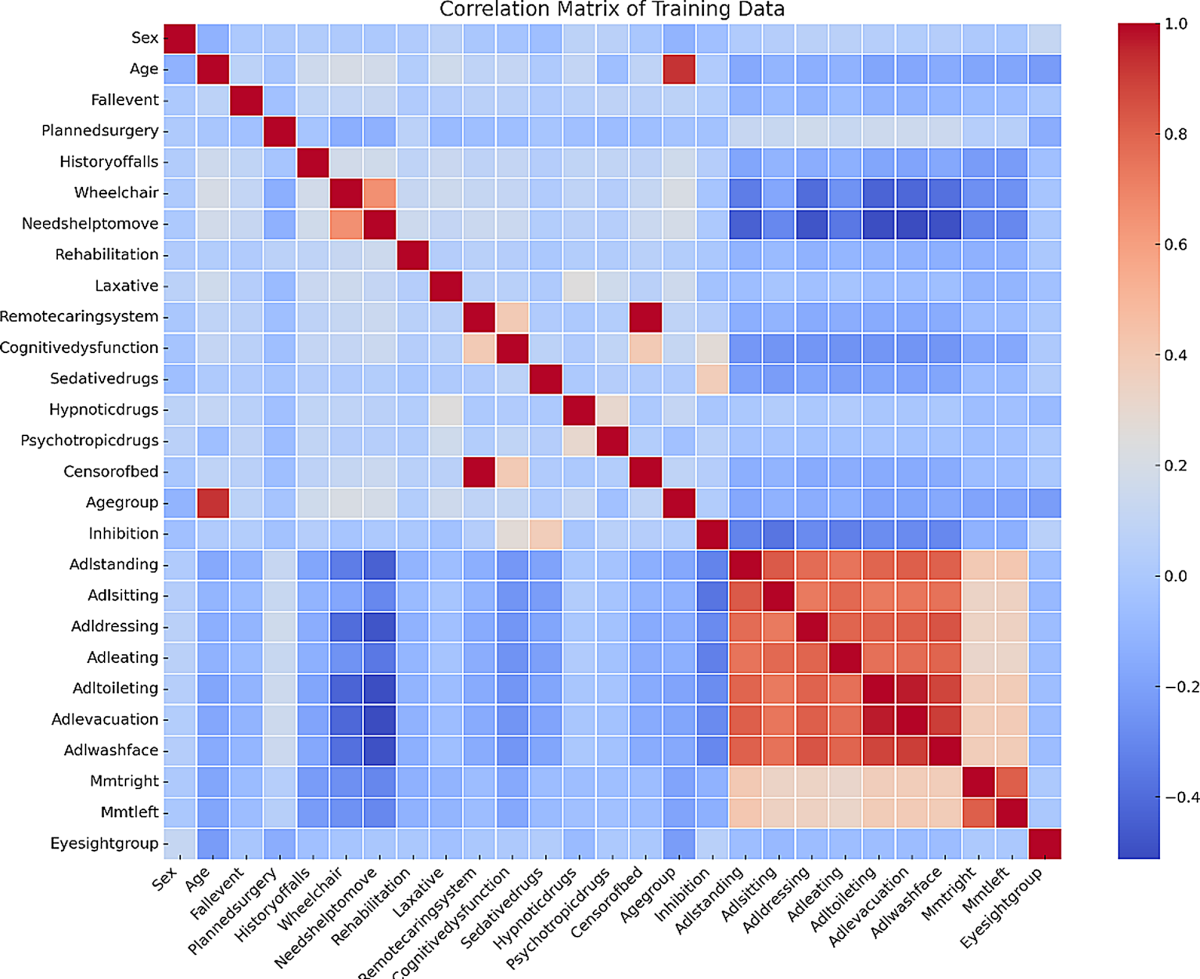
Correlation matrix heat map showing the linear relationships between variables. ADL_related variables (e.g., Adl_standing, Adl_sitting, Adl_dressing) display strong positive correlations, suggesting redundancy. Features with weak correlations (e.g., Sex, Rehabilitation, Sedative_drugs) likely have limited impact on prediction. The strong correlations between Adl_sitting, Adl_dressing, and Adleating highlight potential multicollinearity, warranting the removal of redundant features

Heatmaps were employed to visualize correlations between variables, while a correlation matrix was used to identify potential multicollinearity issues among features. Highly correlated variables—those with a correlation coefficient approaching 1 or -1—indicate potential covariance. For instance, the strong correlations observed among certain ADL-related variables (e.g., Adl_sitting, Adl_dressing, Adl_eating) suggest they may convey similar information. Figure 2 presents the heat map of variable correlations.

To address covariance, we managed multicollinearity by removing redundant variables. In instances where multiple variables showed high correlations (e.g., the red zones among ADL-related variables), certain redundant features were excluded to mitigate the impact of multicollinearity. Additionally, univariate analyses were performed to eliminate non-significant variables, using chi-square tests for categorical variables to assess whether differences between the fall and non-fall groups were statistically significant. As a result of this process, the following variables were removed: Age, Adl_sitting, Adl_dressing, Adl_eating, Needs_help_to_move, Censor_of_bed, Sex, Rehabilitation, Sedative_drugs, Inhibition, and Eyesight_group.

To identify the factors that contribute to fall risk among hospitalized patients and to optimize the model parameters, a binomial Lasso regression was applied to the training dataset, utilizing the SMOTE-ENN technique for balancing. The tuning parameter (λ) was selected through cross-validation to determine the optimal value that minimized the prediction error. The λ value corresponding to the minimum mean squared error was 0.004, and the λ value for the standard error of the minimum distance was 0.022. This process yielded a total of 9 variables that were deemed significant for the model: ‘Age group’, ‘Adl_standing’, ‘Adl_evacuation’, ‘History of falls’, ‘Wheelchair’, ‘Psychotropic_drugs’, ‘Hypnotic_drugs’, ‘Planned surgery’, and ‘Remote caring system’.

**Fig. 3:**
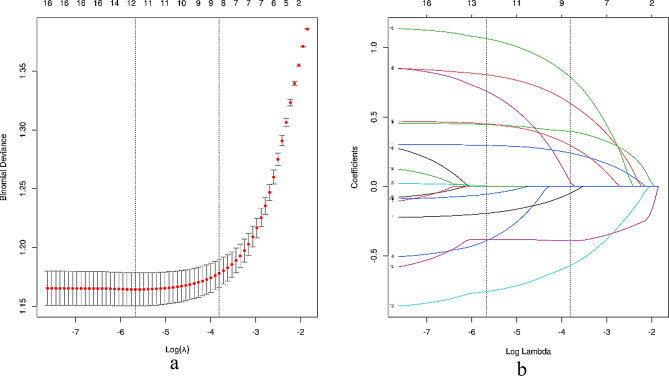
Screening of characterization factors using Lasso regression analysis. (**a**) The plot displays vertical lines representing the lambda values selected through 10-fold cross-validation. The optimal lambda resulted in 9 nonzero coefficients, highlighting the importance of these variables in the predictive model. (**b**) The distribution of coefficients for the 16 texture features is depicted across the log(λ) series in the Lasso regression model. Vertical dashed lines indicate the minimum mean square error (λ = 0.004), corresponding to the lambda that minimizes cross-validation error, and the standard error of the minimum distance (λ = 0.022), which identifies a reduced set of 9 variables. This streamlined approach enhances model interpretability while minimizing the risk of overfitting, offering key insights into the regularization and parameter selection process

The coefficient profiles of the Lasso regression analysis are displayed in Fig. 3a, highlighting the importance and influence of each variable. Additionally, the cross-validation profile is presented in Fig. 3b, illustrating the model’s performance in terms of regularization and parameter selection.

### Synthesis of categorized multi-models

To accomplish the classification task of the data samples, we employed eight mainstream machine learning models: Extreme Gradient Boosting (XGBoost), Logistic Regression(LR), Random Forest(RF), Gradient Boosting Decision Trees (GBDT), Gaussian Plain Bayes (GNB), Multi-Layer Perceptron (MLP), Support Vector Machine (SVM), and K-Nearest Neighbors (KNN). The validation method involved cross-validation with a validation set in a ratio of 8:2 using the random number method, which was trained and repeated five times. The model parameters included a regularization factor of 1, a convergence measure of 0.0001, 100 iterations, and a selected regularization type of 12. The predictive performance of the models was evaluated using the AUC values.

The AUC, cutoff, accuracy, sensitivity, specificity, positive predictive value, negative predictive value, F1-score, and Kappa value for the multi-model classification in the training set and validation set are summarized in Supplementary Tables S6.

Model Comparison: All machine learning models were evaluated using the same set of 9 variables identified in the refined variable selection process. This ensures a fair comparison and reflects the true performance of each model. The Random Forest model, along with other models such as GBDT and XGBoost, demonstrated strong predictive performance with improved sensitivity and F1-scores, indicating a balanced approach to variable selection and model evaluation.

**Fig. 4:**
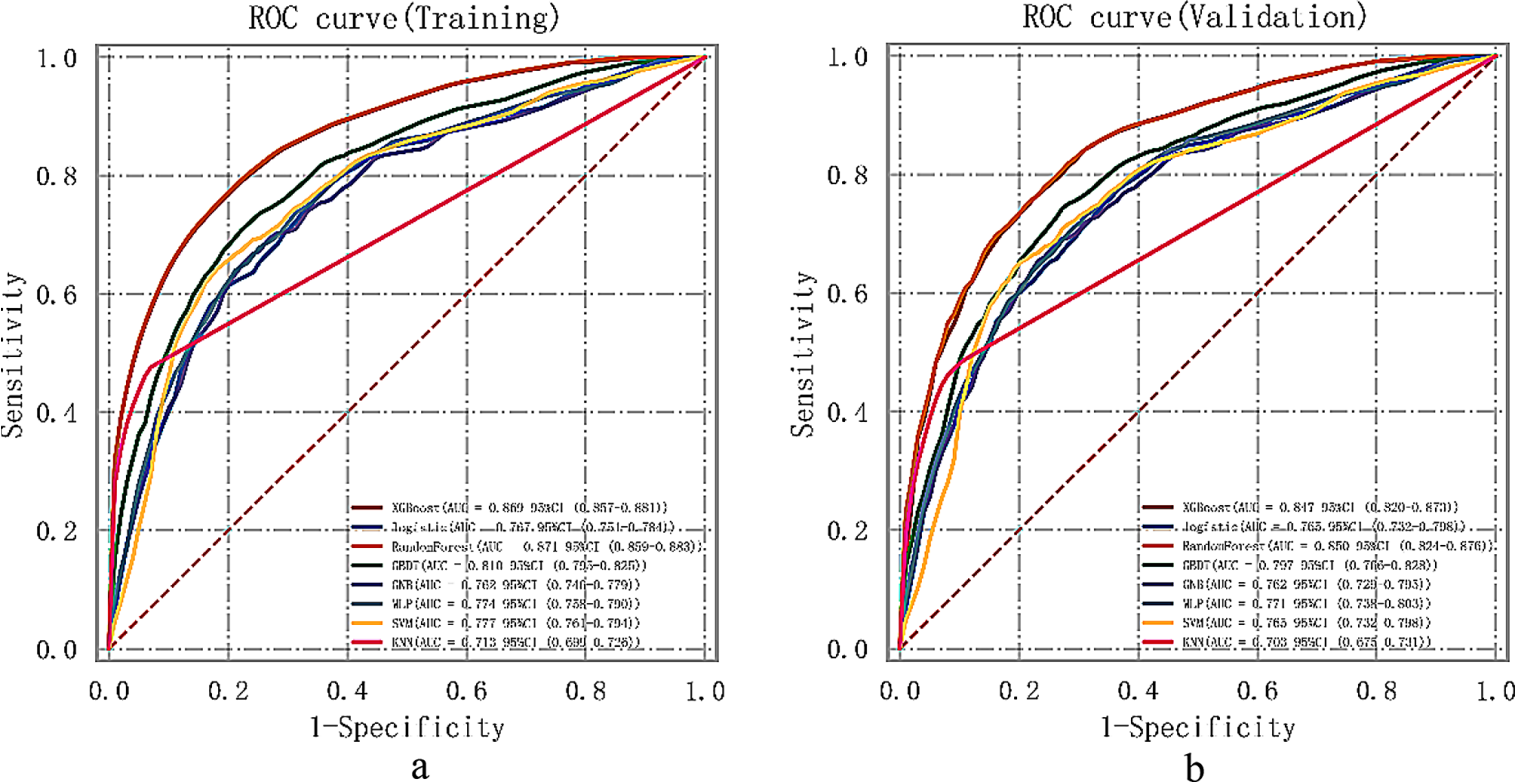
Analysis of ML Model Synthesis. (**a**) ROC curve and AUC for the training set. (**b**) ROC curve and AUC for the validation set. The hospitalized patients dataset was divided into a training set and a validation set in a ratio of 8:2 using the random number method. The cross-validation process was repeated five times to ensure robustness and reliability in the analysis

Among the current models, the Random Forest model performed the best based on the AUC values in the training set, a comprehensive analysis suggests that the Random Forest model can be considered as the optimal choice. The comprehensive analysis of the machine learning multi-model is presented in Fig. 4.

### Optimal model construction and evaluation

To further analyze the data, the Random Forest machine learning method was employed. A test set comprising *N* = 1,894 cases (20% of the total sample) was randomly selected, while the remaining samples were utilized as the training set for 10-fold cross-validation. The model parameters were set as follows: C (regularization factor) = 1.0, max_iter (number of iterations) = 100, penalty (type of regularization) = l2, and tol (convergence measure) = 0.0001.

**Fig. 5:**
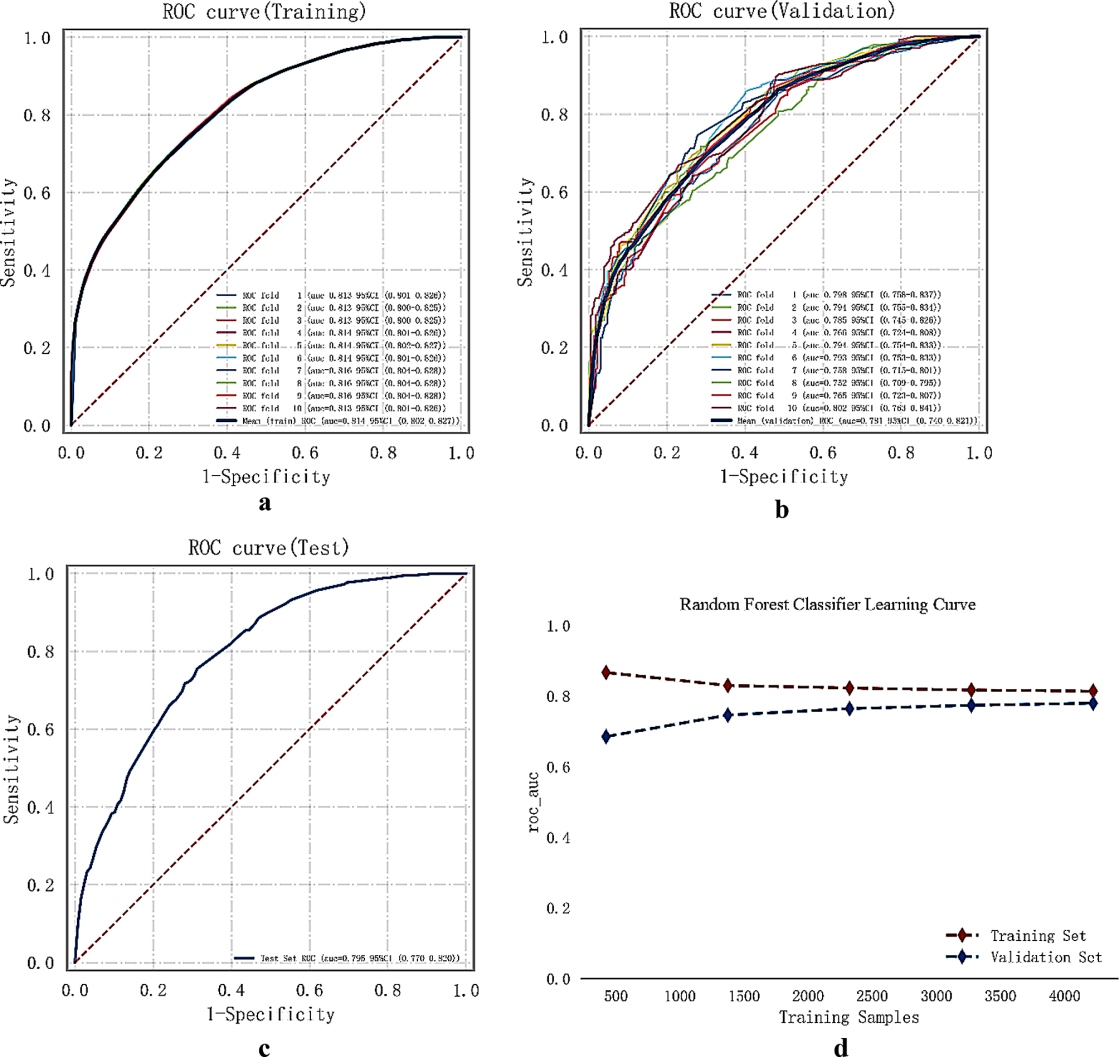
Training, Validation, and Test of the Random Forest Machine Learning Model. (**a**) ROC curve and AUC for the training set. (**b**) ROC curve and AUC for the validation set. The model was trained and cross-validated using 80% of the patients. (**c**) ROC curve and AUC for the test set. The model was tested on another 20% of the patients. (**d**) Learning curve. The red dashed line represents the training set, and the blue dashed line represents the validation set. The ratio of the validation set to the test set, measured by the AUC index, was less than 10%, indicating successful model fitting. The learning curve demonstrates the high stability of the training and validation sets

The results demonstrated an average AUC of 0.814(0.802–0.827) in the training set, 0.781(0.740–0.821) in the validation set, and 0.795 (0.770–0.820) in the test set (Fig. 5a-c). The stability of the model predictions was observed as the AUC values for the training, validation, and test sets stabilized around 0.8. The successful fit of the model was confirmed, as the performance of the validation set did not surpass that of the test set or exceed the 10% threshold. The learning curves further indicated a strong fit and high stability between the training and validation sets (Fig. 5d).

Based on these results, the Random Forest model can be considered suitable for the classification modeling task using this dataset. The AUC, cutoff, accuracy, sensitivity, specificity, positive predictive value, negative predictive value, F1-score, and kappa value for the training set, validation set, and test set using the Random Forest machine learning method are summarized in Supplementary Table S7. The definitions and interpretations of the performance metrics used in the machine learning classification task are presented in Supplementary Table S8. Calibration plot and decision Curve are shown in Supplementary Figures S1.

### SHAP for model interpretation and importance ranking

**Fig. 6:**
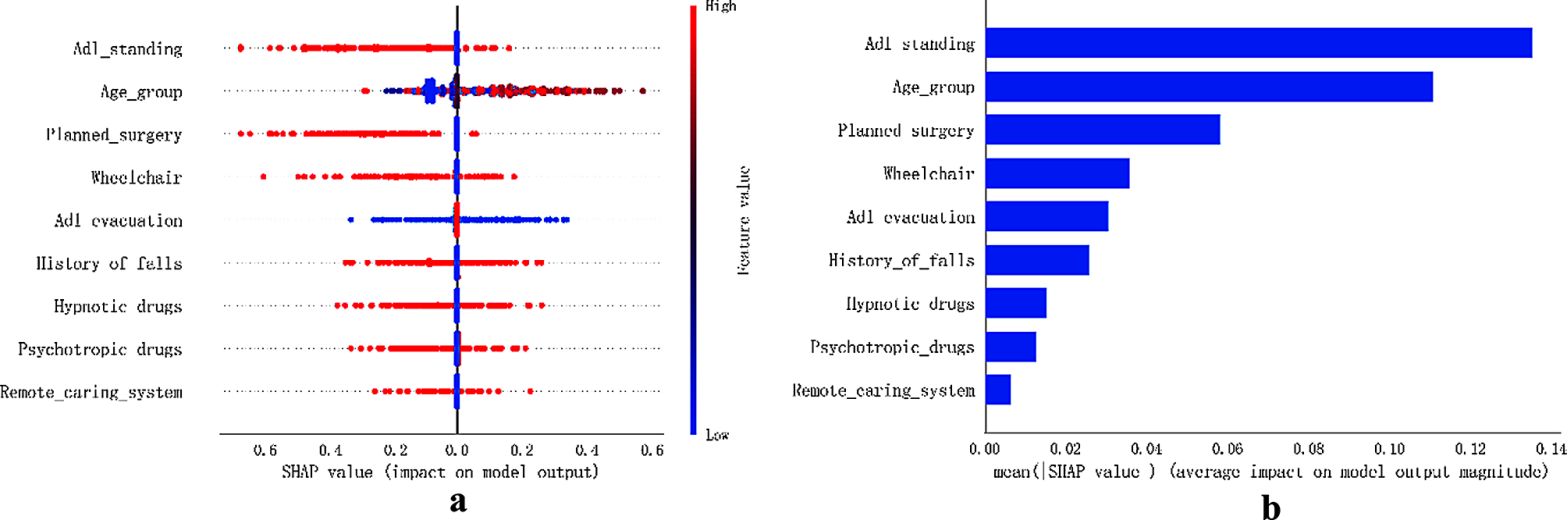
SHAP Interpretation Model. (**a**) SHAP Plot for Feature Contributions. High SHAP values (red) indicate higher risk, while low values (blue) suggest reduced fall risk. (**b**) Ranking of Predictor Importance. Feature ranking based on LASSO regression results

we employed SHAP (SHapley Additive exPlanations) to enhance the interpretability and validation of our model. SHAP values offer a visual and intuitive understanding of the contribution of each feature to individual predictions. This approach ensures consistency in interpretation across different models and aids in validating the model’s behavior, which is crucial for clinical applications. Figure 6 displays the 9 most important features in our model. Each feature is represented by a significant line, with patient attributions to outcomes depicted as colored dots. Red dots indicate high-risk values, while blue dots represent low-risk values. The analysis revealed that factors such as advanced age, Wheelchair use, ADL_evacuation, Fall history, Hypnotic, and Psychotropic medications, etc. increase the risk of falls in hospitalized patients. Conversely, the risk of falls decreases in patients scheduled for surgery (Fig. 6a).

Additionally, we performed variable importance analysis using the Lasso regression model with five-fold cross-validation, 1000 iterations, and a convergence measure of 0.0001. The order of importance for the predictors is depicted in Fig. 6b.

## Discussion

In-hospital falls have significant implications, including adverse outcomes and increased healthcare costs. As a result, numerous studies have explored the risk factors associated with these falls. For instance, a study involving 161 elderly patients utilized a self-report questionnaire and identified several risk factors for in-hospital falls, including history of previous falls, decreased self-care, sleep disorders, hearing impairments, excessive spinal kyphosis, chronic illnesses, platelet count, left rectus femoris muscle thickness (LF-MLT), and left rectus femoris muscle cross-sectional area (LF-CSA). Based on these factors, the study developed a fall prediction model with good predictive efficiency (AUC = 0.920). However, this model required hematological indices and musculoskeletal ultrasound, rendering it less conducive to early assessment of fall risk upon admission to the hospital [13]. Another Japanese study focusing on in-hospital falls in older hospitalized adults demonstrated that malnutrition upon admission was a significant risk factor, with malnourished patients having a 2.7 times higher risk of in-hospital falls compared to non-malnourished patients [14]. Gender also appears to play a role in fall occurrence. A systematic evaluation of fall-related injuries in Chinese older adults concluded that women were at a higher risk of falls, whereas another study involving 1,000 older adults found similar results [15]. However, it is worth noting that the latter study primarily focused on a community-based residential setting. Consequently, further research is warranted to investigate the association between in-hospital falls and gender. Our present study indicated that the incidence of in-hospital falls does not significantly differ between sexes, suggesting that both males and females face an equal risk of experiencing such falls. Additionally, prior research has consistently shown that the use of specific medications increases the likelihood of in-hospital falls. For example, an Australian retrospective study reported a 2.2-fold increase in the incidence of falls among patients with dementia using anticholinergic medications, as well as a 3.1-fold increase in falls with concurrent daily use of multiple psychotropic medications [2]. Our findings align with these previous studies.

Traditionally, the Morse Falls Scale has long been utilized as a comprehensive tool for assessing fall risk in clinical practice [16, 17]. It presents several advantages, such as simplicity, rapid assessment, and the ability to assist in risk stratification. However, there are limitations associated with its use, which can be summarized as follows: Limited scope of application: The Morse Falls Scale is primarily designed for use in older adults and hospitalized patients, and its accuracy in assessing the risk of falling in other populations may be limited.

Lack of consideration for specific risk factors: The Morse Falls Scale focuses mainly on common risk factors for falling and does not take into account other important factors, such as vertigo, visual impairment, cognitive decline, and more. Limited predictive power: The Morse Falls Scale provides a relative assessment of a patient’s risk level for falls but does not accurately predict the occurrence of specific fall events [18]. As a result, its predictive capability is limited. While the Morse Falls Scale has been widely adopted for fall risk assessment, these limitations emphasize the need for alternative approaches that can overcome these shortcomings and provide more accurate and comprehensive risk prediction.

To enhance and refine the previous in-hospital fall risk assessment system, this study aimed to utilize machine learning algorithms to develop a prediction model that identifies important fall risk factors for early assessment and intervention upon admission. The study population consisted of 9,470 hospitalized patients, among whom 230 experienced a fall event, resulting in an incidence rate of 2.43%, which aligns with findings from a multicenter retrospective study [19]. We utilized the Synthetic Minority Over-sampling Technique and Edited Nearest Neighbors (SMOTE-ENN) to balance the training set data. The dataset was subsequently analyzed using univariate analyses and Lasso regression model to select nine key variables from an initial set of 26 clinical factors, which were used to evaluate fall risk in hospitalized patients. These nine variables included Age group, Adl_standing, Adl_evacuation, History of falls, Wheelchair, Psychotropic drugs, Hypnotic drugs, Planned surgery, and Remote caring system, all of which were significantly associated with fall occurrence. Notably, planned surgery was found to be a protective factor against in-hospital falls, suggesting that medical patients face a higher risk compared to surgical patients. This observation may be attributed to better general health among patients with planned surgeries, while patients with medical disorders could be at increased risk due to underlying conditions and advanced age.

Previous studies have summarized the risk factors for in-hospital falls; however, predictive models have not yet been developed [20–23]. In our study, we evaluated eight mainstream machine learning models and analyzed various performance metrics, including AUC, accuracy, sensitivity, specificity, positive predictive value, negative predictive value, and F1- score, for both the training and validation sets. Among the models tested, the AUC of the Random Forest model was 0.814(0.802–0.827) in the training set, 0.781(0.740–0.821) in the validation set, and 0.795 (0.770–0.820) in the testing set, and the results showed that the method had good stability and diagnostic accuracy. Therefore, after a comprehensive evaluation, we chose the Random Forest model for classification. The performance of the Random Forest model was further assessed by comparing the AUC values of the validation set with those of the test set. The results indicated successful fitting, with the validation set either matching or showing a difference of less than 10% compared to the test set. Thus, the Random Forest model was deemed suitable for the classification modeling task in this dataset. Based on the AUC values obtained in our study, the predictive model’s performance and discriminative ability may be superior to previous similar studies [24, 25].

To enhance the comprehensibility and interpretability of the machine prediction model for clinicians, we employed the SHAP (Shapley Additive exPlanations) method in conjunction with the logistic regression model. This integration aimed to improve prediction accuracy and facilitate model interpretation [26]. The SHAP method is a machine learning algorithm specifically designed for model interpretation. It leverages the concept of Shapley values to elucidate the contribution of each feature to the model’s output. By calculating the Shapley value for each feature, the SHAP method quantifies the impact of individual features on the prediction outcome. This enables a comprehensive explanation of the model’s prediction results. The underlying principle involves computing the Shapley value through permutations of feature values. The Shapley value, derived from game theory, measures the contribution of each feature to the prediction outcome. Summing the Shapley values across all features yields a global feature importance score, providing a holistic understanding of the model’s prediction outcomes. By employing the SHAP methodology, we can elucidate the impact of each feature in the model on the predicted results, as well as capture the interplay between different features. This explanatory approach aids in understanding the decision-making process of the model and reveals the key factors driving the predicted outcomes [27]. The SHAP method finds utility across various predictive models, encompassing regression models, classification models, and more. It can effectively explain the prediction results generated by different machine learning algorithms, such as decision trees, neural networks, and support vector machines [28]. Leveraging the SHAP method allows researchers to gain a better grasp of the models’ predictive capabilities and provide interpretable explanations for the results.

While previous studies have identified fall risk factors like history of falls, cognitive dysfunction, and substance use, our study goes beyond developing a fall risk prediction model using machine learning algorithms. This approach not only replicates the findings of prior research [29] but also adds value by offering an interpretable model. As a result, a more comprehensive and accurate assessment of individual risk is achieved, paving the way for targeted intervention and prevention strategies. Accurately identifying high-risk populations enables healthcare providers to implement tailored interventions, such as heightened supervision, environmental enhancements, and medication adjustments, aimed at preventing falls. Furthermore, health policymakers can rely on the predictive model to inform the development of scoring tools and guidelines for fall prevention within healthcare organizations. Consequently, our study holds positive implications for both clinical practice and public health.

### Limitations

Our study has several limitations that should be acknowledged. Firstly, this was a retrospective study, which introduces the possibility of selection bias. Future prospective studies are necessary to validate the findings. Additionally, further research is needed to assess the impact of implementing the predictive model on fall prevention outcomes and patient safety. External validation is also required to enhance the performance of the predictive model and facilitate its integration into clinical practice. Secondly, the dataset used in our study included a limited number of clinical risk factors. There may be important clinical risk factors that were not included, and the AUC value of the validation and test sets were less than 0.8. Therefore, the optimization of the predictive model could be further enhanced by incorporating more clinical variables. Thirdly, it should be noted that our dataset was derived from a single center. Although the nomogram has been validated in an independent cohort, it is important to recognize that the applicability of the nomogram in different hospitals and countries with diverse patient populations may be limited. In summary, while our study provides valuable insights into fall risk prediction among hospitalized patients, it is crucial to consider these limitations when interpreting the results. Future studies addressing these limitations will contribute to the refinement and broader applicability of the predictive model.

## Conclusions

In conclusion, this study developed a machine learning-based predictive model to assess fall risk in hospitalized patients. The Random Forest model showcased excellent performance. The identification of specific risk factors emphasizes the importance of early intervention for individuals at high risk of falls. The predictive model serves as a valuable tool for clinicians in identifying and addressing fall occurrences among hospitalized patients. Furthermore, it can aid public health policymakers in the development of improved scoring tools.

## Electronic supplementary material

Below is the link to the electronic supplementary material.

Supplementary Material 1

## Data Availability

No datasets were generated or analysed during the current study.

## Abbreviations

ADL: Activity of Daily Living
AUC: Area Under the ROC Curve
CI: Confidence Interval
FN: False Negative
FP: False Positive
GBDT: Gradient Boosting Decision Trees
GNB: Gaussian Naive Bayes
IQR: Interquartile Range
KNN: K-Nearest Neighbors
LR: Logistic Regression
MFS: Morse Fall Scale
MLP: Multi-Layer Perceptron
NPV: Negative Predictive Value
OR: Odds Ratio
P: P-value
PPV: Positive Predictive Value
RF: Random Forest
ROC: Receiver Operator Characteristic
SE: Standard Error
SHAP: Shapley Additive Explanations
SVM: Support Vector Machine
SMOTE-ENN: Synthetic Minority Oversampling Technique and Edited Nearest Neighbors
TN: True Negative
TP: True Positive
XGBoost: Extreme Gradient Boosting
Z: Z-score
FMUH: Fukushima Medical University Hospital
LASSO: Least Absolute Shrinkage and Selection Operator
