# Supplementary material for: Constructing a fall risk prediction model for hospitalized patients using machine learning
Source: BMC Public Health. 2025 Jan 20;25:242. doi: 10.1186/s12889-025-21284-8 (PMC11748310; doi:10.1186/s12889-025-21284-8)
Supplement: Supplementary file 1 — Supplementary Material 1 [file 12889_2025_21284_MOESM1_ESM.docx]

**Supplementary material:**

**Table S1. Specific baseline data for the training**, **validation and test sets（original data）**

|  | **Group** | | |  |
| --- | --- | --- | --- | --- |
| **Variable** | **Overall**, N = 9,470^1^ | **Training & validation Set**,  N = 7,576^1^ | **Test Set**,  N = 1,894^1^ | **p-value**^2^ |
| **Age, Median (IQR)** | 63.000 (48.000 – 73.000) | 62.000 (49.000 – 73.000) | 63.000 (47.000 – 73.000) | 0.243 |
| (Missing) | 1 | 0 | 1 |  |
| **Sex, n (%)** |  |  |  | 0.934 |
| 1 | 4,748 (50) | 3,800 (50) | 948 (50) |  |
| 2 | 4,722 (50) | 3,776 (50) | 946 (50) |  |
| **Fallevent, n (%)** |  |  |  | 0.617 |
| 0 | 9,240 (98) | 7,389 (98) | 1,851 (98) |  |
| 1 | 230 (2.4) | 187 (2.5) | 43 (2.3) |  |
| **Planned surgery, n (%)** |  |  |  | 0.066 |
| 0 | 5,475 (58) | 4,416 (59) | 1,059 (57) |  |
| 1 | 3,903 (42) | 3,088 (41) | 815 (43) |  |
| (Missing) | 92 | 72 | 20 |  |
| **History of falls, n (%)** |  |  |  | 0.680 |
| 0 | 8,631 (91) | 6,901 (91) | 1,730 (91) |  |
| 1 | 828 (8.8) | 667 (8.8) | 161 (8.5) |  |
| (Missing) | 11 | 8 | 3 |  |
| **Wheelchair, n (%)** |  |  |  | 0.534 |
| 0 | 7,601 (80) | 6,071 (80) | 1,530 (81) |  |
| 1 | 1,868 (20) | 1,504 (20) | 364 (19) |  |
| (Missing) | 1 | 1 | 0 |  |
| **Needs help to move, n (%)** |  |  |  | 0.096 |
| 0 | 8,127 (86) | 6,524 (86) | 1,603 (85) |  |
| 1 | 1,342 (14) | 1,051 (14) | 291 (15) |  |
| (Missing) | 1 | 1 | 0 |  |
| **Rehabilitation, n (%)** |  |  |  | 0.115 |
| 0 | 9,216 (97) | 7,362 (97) | 1,854 (98) |  |
| 1 | 249 (2.6) | 209 (2.8) | 40 (2.1) |  |
| (Missing) | 5 | 5 | 0 |  |
| **Laxative, n (%)** |  |  |  | 0.177 |
| 0 | 7,800 (82) | 6,219 (82) | 1,581 (83) |  |
| 1 | 1,664 (18) | 1,351 (18) | 313 (17) |  |
| (Missing) | 6 | 6 | 0 |  |
| **Remote caring system, n (%)** |  |  |  | 0.772 |
| 0 | 9,391 (99) | 7,512 (99) | 1,879 (99) |  |
| 1 | 75 (0.8) | 61 (0.8) | 14 (0.7) |  |
| (Missing) | 4 | 3 | 1 |  |
| **Cognitive dysfunction, n (%)** |  |  |  | 0.823 |
| 0 | 9,231 (97) | 7,385 (97) | 1,846 (98) |  |
| 1 | 237 (2.5) | 191 (2.5) | 46 (2.4) |  |
| (Missing) | 2 | 0 | 2 |  |
| **Sedative drugs, n (%)** |  |  |  | 0.012 |
| 0 | 9,270 (98) | 7,430 (98) | 1,840 (97) |  |
| 1 | 200 (2.1) | 146 (1.9) | 54 (2.9) |  |
| **Hypnotic drugs, n (%)** |  |  |  | 0.701 |
| 0 | 8,003 (85) | 6,397 (84) | 1,606 (85) |  |
| 1 | 1,467 (15) | 1,179 (16) | 288 (15) |  |
| **Psychotropic drugs, n (%)** |  |  |  | 0.816 |
| 0 | 8,863 (94) | 7,088 (94) | 1,775 (94) |  |
| 1 | 606 (6.4) | 487 (6.4) | 119 (6.3) |  |
| (Missing) | 1 | 1 | 0 |  |
| **Censor of bed, n (%)** |  |  |  | 0.772 |
| 0 | 9,391 (99) | 7,512 (99) | 1,879 (99) |  |
| 1 | 75 (0.8) | 61 (0.8) | 14 (0.7) |  |
| (Missing) | 4 | 3 | 1 |  |
| **Age group, n (%)** |  |  |  | 0.063 |
| 1 | 2,501 (26) | 1,971 (26) | 530 (28) |  |
| 2 | 2,538 (27) | 2,071 (27) | 467 (25) |  |
| 3 | 2,440 (26) | 1,934 (26) | 506 (27) |  |
| 4 | 1,990 (21) | 1,600 (21) | 390 (21) |  |
| (Missing) | 1 | 0 | 1 |  |
| **Inhibition, n (%)** |  |  |  | 0.065 |
| 0 | 9,267 (98) | 7,424 (98) | 1,843 (97) |  |
| 1 | 203 (2.1) | 152 (2.0) | 51 (2.7) |  |
| **Adl**_**standing, n (%)** |  |  |  | 0.121 |
| 0 | 1,480 (16) | 1,162 (15) | 318 (17) |  |
| 1 | 7,987 (84) | 6,411 (85) | 1,576 (83) |  |
| (Missing) | 3 | 3 | 0 |  |
| **Adl**_**sitting, n (%)** |  |  |  | 0.023 |
| 0 | 1,088 (11) | 842 (11) | 246 (13) |  |
| 1 | 8,377 (89) | 6,729 (89) | 1,648 (87) |  |
| (Missing) | 5 | 5 | 0 |  |
| **Adl**_**dressing, n (%)** |  |  |  | 0.039 |
| 0 | 1,763 (19) | 1,379 (18) | 384 (20) |  |
| 1 | 7,704 (81) | 6,194 (82) | 1,510 (80) |  |
| (Missing) | 3 | 3 | 0 |  |
| **Adl**_**eating, n (%)** |  |  |  | 0.017 |
| 0 | 1,371 (14) | 1,064 (14) | 307 (16) |  |
| 1 | 8,096 (86) | 6,509 (86) | 1,587 (84) |  |
| (Missing) | 3 | 3 | 0 |  |
| **Adl**_**toileting, n (%)** |  |  |  | 0.113 |
| 0 | 1,789 (19) | 1,407 (19) | 382 (20) |  |
| 1 | 7,675 (81) | 6,164 (81) | 1,511 (80) |  |
| (Missing) | 6 | 5 | 1 |  |
| **Adl**_**evacuation, n (%)** |  |  |  | 0.077 |
| 0 | 1,741 (18) | 1,366 (18) | 375 (20) |  |
| 1 | 7,726 (82) | 6,207 (82) | 1,519 (80) |  |
| (Missing) | 3 | 3 | 0 |  |
| **Adl**_**washface, n (%)** |  |  |  | 0.191 |
| 0 | 1,638 (17) | 1,291 (17) | 347 (18) |  |
| 1 | 7,828 (83) | 6,281 (83) | 1,547 (82) |  |
| (Missing) | 4 | 4 | 0 |  |
| **Mmtright, n (%)** |  |  |  | 0.264 |
| 0 | 777 (8.3) | 634 (8.4) | 143 (7.6) |  |
| 1 | 8,638 (92) | 6,904 (92) | 1,734 (92) |  |
| (Missing) | 55 | 38 | 17 |  |
| **Mmtleft, n (%)** |  |  |  | 0.526 |
| 0 | 796 (8.4) | 644 (8.5) | 152 (8.1) |  |
| 1 | 8,625 (92) | 6,897 (91) | 1,728 (92) |  |
| (Missing) | 49 | 35 | 14 |  |
| **Eyesight group, n (%)** |  |  |  | 0.407 |
| 0 | 4,502 (48) | 3,618 (48) | 884 (47) |  |
| 1 | 4,956 (52) | 3,949 (52) | 1,007 (53) |  |
| (Missing) | 12 | 9 | 3 |  |
| ^1^Median (IQR) or Frequency (%) | | | | |
| ^2^Wilcoxon rank sum test; Pearson's Chi-squared test | | | | |

**Table S2. Description of the results of training & validation set data analysis （original data）**

|  | **Group** | | |  |
| --- | --- | --- | --- | --- |
| **Variable** | **Overall**, N = 7,576^1^ | **Fall event=NO**,  N = 7,389^1^ | **Fall event=YES**,  N = 187^1^ | **p-value**^2^ |
| **Age, Median (IQR)** | 62.000 (49.000 – 73.000) | 62.000 (48.000 – 73.000) | 72.000 (60.500 – 78.000) | <0.001 |
| **Sex, n (%)** |  |  |  | 0.574 |
| 1 | 3,800 (50) | 3,710 (50) | 90 (48) |  |
| 2 | 3,776 (50) | 3,679 (50) | 97 (52) |  |
| **Planned surgery, n (%)** |  |  |  | <0.001 |
| 0 | 4,416 (59) | 4,283 (59) | 133 (72) |  |
| 1 | 3,088 (41) | 3,037 (41) | 51 (28) |  |
| (Missing) | 72 | 69 | 3 |  |
| **History of falls, n (%)** |  |  |  | <0.001 |
| 0 | 6,901 (91) | 6,761 (92) | 140 (75) |  |
| 1 | 667 (8.8) | 620 (8.4) | 47 (25) |  |
| (Missing) | 8 | 8 | 0 |  |
| **Wheelchair, n (%)** |  |  |  | <0.001 |
| 0 | 6,071 (80) | 5,971 (81) | 100 (53) |  |
| 1 | 1,504 (20) | 1,417 (19) | 87 (47) |  |
| (Missing) | 1 | 1 | 0 |  |
| **Needs help to move, n (%)** |  |  |  | <0.001 |
| 0 | 6,524 (86) | 6,413 (87) | 111 (59) |  |
| 1 | 1,051 (14) | 975 (13) | 76 (41) |  |
| (Missing) | 1 | 1 | 0 |  |
| **Rehabilitation, n (%)** |  |  |  | 0.083 |
| 0 | 7,362 (97) | 7,184 (97) | 178 (95) |  |
| 1 | 209 (2.8) | 200 (2.7) | 9 (4.8) |  |
| (Missing) | 5 | 5 | 0 |  |
| **Laxative, n (%)** |  |  |  | <0.001 |
| 0 | 6,219 (82) | 6,084 (82) | 135 (72) |  |
| 1 | 1,351 (18) | 1,299 (18) | 52 (28) |  |
| (Missing) | 6 | 6 | 0 |  |
| **Remote caring system, n (%)** |  |  |  | <0.001 |
| 0 | 7,512 (99) | 7,333 (99) | 179 (96) |  |
| 1 | 61 (0.8) | 53 (0.7) | 8 (4.3) |  |
| (Missing) | 3 | 3 | 0 |  |
| **Cognitive dysfunction, n (%)** |  |  |  | <0.001 |
| 0 | 7,385 (97) | 7,214 (98) | 171 (91) |  |
| 1 | 191 (2.5) | 175 (2.4) | 16 (8.6) |  |
| **Sedative drugs, n (%)** |  |  |  | 0.094 |
| 0 | 7,430 (98) | 7,250 (98) | 180 (96) |  |
| 1 | 146 (1.9) | 139 (1.9) | 7 (3.7) |  |
| **Hypnotic drugs, n (%)** |  |  |  | <0.001 |
| 0 | 6,397 (84) | 6,264 (85) | 133 (71) |  |
| 1 | 1,179 (16) | 1,125 (15) | 54 (29) |  |
| **Psychotropic drugs, n (%)** |  |  |  | <0.001 |
| 0 | 7,088 (94) | 6,936 (94) | 152 (81) |  |
| 1 | 487 (6.4) | 452 (6.1) | 35 (19) |  |
| (Missing) | 1 | 1 | 0 |  |
| **Censor of bed, n (%)** |  |  |  | <0.001 |
| 0 | 7,512 (99) | 7,333 (99) | 179 (96) |  |
| 1 | 61 (0.8) | 53 (0.7) | 8 (4.3) |  |
| (Missing) | 3 | 3 | 0 |  |
| **Age group, n (%)** |  |  |  | <0.001 |
| 1 | 1,971 (26) | 1,945 (26) | 26 (14) |  |
| 2 | 2,071 (27) | 2,042 (28) | 29 (16) |  |
| 3 | 1,934 (26) | 1,872 (25) | 62 (33) |  |
| 4 | 1,600 (21) | 1,530 (21) | 70 (37) |  |
| **Inhibition, n (%)** |  |  |  | 0.013 |
| 0 | 7,424 (98) | 7,246 (98) | 178 (95) |  |
| 1 | 152 (2.0) | 143 (1.9) | 9 (4.8) |  |
| **Adl**_**standing, n (%)** |  |  |  | <0.001 |
| 0 | 1,162 (15) | 1,085 (15) | 77 (41) |  |
| 1 | 6,411 (85) | 6,301 (85) | 110 (59) |  |
| (Missing) | 3 | 3 | 0 |  |
| **Adl**_**sitting, n (%)** |  |  |  | <0.001 |
| 0 | 842 (11) | 795 (11) | 47 (25) |  |
| 1 | 6,729 (89) | 6,589 (89) | 140 (75) |  |
| (Missing) | 5 | 5 | 0 |  |
| **Adl**_**dressing, n (%)** |  |  |  | <0.001 |
| 0 | 1,379 (18) | 1,295 (18) | 84 (45) |  |
| 1 | 6,194 (82) | 6,091 (82) | 103 (55) |  |
| (Missing) | 3 | 3 | 0 |  |
| **Adl**_**eating, n (%)** |  |  |  | <0.001 |
| 0 | 1,064 (14) | 1,008 (14) | 56 (30) |  |
| 1 | 6,509 (86) | 6,378 (86) | 131 (70) |  |
| (Missing) | 3 | 3 | 0 |  |
| **Adl**_**toileting, n (%)** |  |  |  | <0.001 |
| 0 | 1,407 (19) | 1,321 (18) | 86 (46) |  |
| 1 | 6,164 (81) | 6,063 (82) | 101 (54) |  |
| (Missing) | 5 | 5 | 0 |  |
| **Adl**_**evacuation, n (%)** |  |  |  | <0.001 |
| 0 | 1,366 (18) | 1,280 (17) | 86 (46) |  |
| 1 | 6,207 (82) | 6,106 (83) | 101 (54) |  |
| (Missing) | 3 | 3 | 0 |  |
| **Adl**_**washface, n (%)** |  |  |  | <0.001 |
| 0 | 1,291 (17) | 1,213 (16) | 78 (42) |  |
| 1 | 6,281 (83) | 6,172 (84) | 109 (58) |  |
| (Missing) | 4 | 4 | 0 |  |
| **Mmtright, n (%)** |  |  |  | <0.001 |
| 0 | 634 (8.4) | 595 (8.1) | 39 (21) |  |
| 1 | 6,904 (92) | 6,756 (92) | 148 (79) |  |
| (Missing) | 38 | 38 | 0 |  |
| **Mmtleft, n (%)** |  |  |  | <0.001 |
| 0 | 644 (8.5) | 606 (8.2) | 38 (20) |  |
| 1 | 6,897 (91) | 6,749 (92) | 148 (80) |  |
| (Missing) | 35 | 34 | 1 |  |
| **Eyesight group, n (%)** |  |  |  | 0.293 |
| 0 | 3,618 (48) | 3,522 (48) | 96 (52) |  |
| 1 | 3,949 (52) | 3,859 (52) | 90 (48) |  |
| (Missing) | 9 | 8 | 1 |  |
| ^1^Median (IQR) or Frequency (%) | | | | |
| ^2^Wilcoxon rank sum test; Pearson's Chi-squared test; Fisher's exact test | | | | |

**Table S3. Description of the results of training set data analysis using SMOTE-ENN**

|  | **Group** | | |  |
| --- | --- | --- | --- | --- |
| **Variable** | **Overall**, N = 4,000^1^ | **Fall event=NO**,  N = 2,000^1^ | **Fall event=YES**,  N = 2,000^1^ | **p-value**^2^ |
| **Age, Median (IQR)** | 68.000 (54.000 – 76.000) | 63.000 (48.000 – 73.000) | 72.000 (62.000 – 78.000) | <0.001 |
| **Sex, n (%)** |  |  |  | 0.054 |
| 1 | 1,955 (49) | 1,008 (50) | 947 (47) |  |
| 2 | 2,045 (51) | 992 (50) | 1,053 (53) |  |
| **Planned surgery, n (%)** |  |  |  | <0.001 |
| 0 | 2,669 (67) | 1,213 (61) | 1,456 (73) |  |
| 1 | 1,331 (33) | 787 (39) | 544 (27) |  |
| **History of falls, n (%)** |  |  |  | <0.001 |
| 0 | 3,305 (83) | 1,828 (91) | 1,477 (74) |  |
| 1 | 695 (17) | 172 (8.6) | 523 (26) |  |
| **Wheelchair, n (%)** |  |  |  | <0.001 |
| 0 | 2,675 (67) | 1,616 (81) | 1,059 (53) |  |
| 1 | 1,325 (33) | 384 (19) | 941 (47) |  |
| **Needs help to move, n (%)** |  |  |  | <0.001 |
| 0 | 2,909 (73) | 1,739 (87) | 1,170 (59) |  |
| 1 | 1,091 (27) | 261 (13) | 830 (42) |  |
| **Rehabilitation, n (%)** |  |  |  | <0.001 |
| 0 | 3,844 (96) | 1,944 (97) | 1,900 (95) |  |
| 1 | 156 (3.9) | 56 (2.8) | 100 (5.0) |  |
| **Laxative, n (%)** |  |  |  | <0.001 |
| 0 | 3,105 (78) | 1,669 (83) | 1,436 (72) |  |
| 1 | 895 (22) | 331 (17) | 564 (28) |  |
| **Remote caring system, n (%)** |  |  |  | <0.001 |
| 0 | 3,908 (98) | 1,987 (99) | 1,921 (96) |  |
| 1 | 92 (2.3) | 13 (0.7) | 79 (4.0) |  |
| **Cognitive dysfunction, n (%)** |  |  |  | <0.001 |
| 0 | 3,787 (95) | 1,953 (98) | 1,834 (92) |  |
| 1 | 213 (5.3) | 47 (2.4) | 166 (8.3) |  |
| **Sedative drugs, n (%)** |  |  |  | 0.015 |
| 0 | 3,900 (98) | 1,962 (98) | 1,938 (97) |  |
| 1 | 100 (2.5) | 38 (1.9) | 62 (3.1) |  |
| **Hypnotic drugs, n (%)** |  |  |  | <0.001 |
| 0 | 3,130 (78) | 1,701 (85) | 1,429 (71) |  |
| 1 | 870 (22) | 299 (15) | 571 (29) |  |
| **Psychotropic drugs, n (%)** |  |  |  | <0.001 |
| 0 | 3,545 (89) | 1,885 (94) | 1,660 (83) |  |
| 1 | 455 (11) | 115 (5.8) | 340 (17) |  |
| **Censor of bed, n (%)** |  |  |  | <0.001 |
| 0 | 3,908 (98) | 1,987 (99) | 1,921 (96) |  |
| 1 | 92 (2.3) | 13 (0.7) | 79 (4.0) |  |
| **Age group, n (%)** |  |  |  | <0.001 |
| 1 | 788 (20) | 538 (27) | 250 (13) |  |
| 2 | 840 (21) | 526 (26) | 314 (16) |  |
| 3 | 1,185 (30) | 513 (26) | 672 (34) |  |
| 4 | 1,187 (30) | 423 (21) | 764 (38) |  |
| **Inhibition, n (%)** |  |  |  | <0.001 |
| 0 | 3,858 (96) | 1,954 (98) | 1,904 (95) |  |
| 1 | 142 (3.6) | 46 (2.3) | 96 (4.8) |  |
| **Adl**_**standing, n (%)** |  |  |  | <0.001 |
| 0 | 1,125 (28) | 293 (15) | 832 (42) |  |
| 1 | 2,875 (72) | 1,707 (85) | 1,168 (58) |  |
| **Adl**_**sitting, n (%)** |  |  |  | <0.001 |
| 0 | 721 (18) | 226 (11) | 495 (25) |  |
| 1 | 3,279 (82) | 1,774 (89) | 1,505 (75) |  |
| **Adl**_**dressing, n (%)** |  |  |  | <0.001 |
| 0 | 1,230 (31) | 353 (18) | 877 (44) |  |
| 1 | 2,770 (69) | 1,647 (82) | 1,123 (56) |  |
| **Adl**_**eating, n (%)** |  |  |  | <0.001 |
| 0 | 865 (22) | 279 (14) | 586 (29) |  |
| 1 | 3,135 (78) | 1,721 (86) | 1,414 (71) |  |
| **Adl**_**toileting, n (%)** |  |  |  | <0.001 |
| 0 | 1,305 (33) | 363 (18) | 942 (47) |  |
| 1 | 2,695 (67) | 1,637 (82) | 1,058 (53) |  |
| **Adl**_**evacuation, n (%)** |  |  |  | <0.001 |
| 0 | 1,286 (32) | 351 (18) | 935 (47) |  |
| 1 | 2,714 (68) | 1,649 (82) | 1,065 (53) |  |
| **Adl**_**washface, n (%)** |  |  |  | <0.001 |
| 0 | 1,179 (29) | 328 (16) | 851 (43) |  |
| 1 | 2,821 (71) | 1,672 (84) | 1,149 (57) |  |
| **Mmtright, n (%)** |  |  |  | <0.001 |
| 0 | 595 (15) | 170 (8.5) | 425 (21) |  |
| 1 | 3,405 (85) | 1,830 (92) | 1,575 (79) |  |
| **Mmtleft, n (%)** |  |  |  | <0.001 |
| 0 | 608 (15) | 167 (8.4) | 441 (22) |  |
| 1 | 3,392 (85) | 1,833 (92) | 1,559 (78) |  |
| **Eyesight group, n (%)** |  |  |  | 0.011 |
| 0 | 1,964 (49) | 942 (47) | 1,022 (51) |  |
| 1 | 2,036 (51) | 1,058 (53) | 978 (49) |  |
| ^1^Median (IQR) or Frequency (%) | | | | |
| ^2^Wilcoxon rank sum test; Pearson's Chi-squared test | | | | |

**Table S4. Univariate analysis in the first step of screening for characteristic factors**

| **Variable** | **Test** | **P-Value** |
| --- | --- | --- |
| Sex | Chi-Square | 0.625444722 |
| Planned surgery | Chi-Square | 0.000239332 |
| History of falls | Chi-Square | 4.47E-15 |
| Wheelchair | Chi-Square | 4.97E-20 |
| Needs help to move | Chi-Square | 2.54E-26 |
| Rehabilitation | Chi-Square | 0.131419992 |
| Laxative | Chi-Square | 0.000455974 |
| Remote caring system | Chi-Square | 6.87E-07 |
| Adl_sitting | Chi-Square | 1.42E-09 |
| Adl_dressing | Chi-Square | 2.36E-21 |
| Adl_eating | Chi-Square | 4.73E-10 |
| Adl_toileting | Chi-Square | 4.43E-22 |
| Adl_evacuation | Chi-Square | 2.07E-23 |
| Adl_washface | Chi-Square | 2.66E-19 |
| Mmtright | Chi-Square | 1.23E-09 |
| Mmtleft | Chi-Square | 9.34E-09 |
| Eyesight group | Chi-Square | 0.328974386 |
| Cognitive dysfunction | Chi-Square | 3.50E-07 |
| Sedative drugs | Chi-Square | 0.118770811 |
| Hypnotic drugs | Chi-Square | 6.23E-07 |
| Psychotropic drugs | Chi-Square | 1.15E-11 |
| Censor of bed | Chi-Square | 6.87E-07 |
| Age group | Chi-Square | 1.21E-10 |
| Inhibition | Chi-Square | 0.012161001 |
| Adl_standing | Chi-Square | 9.05E-23 |
| Age | Mann-Whitney U | 8.43E-12 |

**Table S5a. LASSO Selected Variables**

| **Variable names** | **Minimum mean squared error** | **Minimum distance standard error** |
| --- | --- | --- |
| (Intercept) | -2.558 | -1.411 |
| Planned surgery | -0.188 | -0.049 |
| History of falls | 0.799 | 0.597 |
| Wheelchair | 0.424 | 0.396 |
| Laxative | 0.0 | 0.0 |
| Remote caring system | 0.69 | 0.029 |
| Cognitive dysfunction | 0.0 | 0.0 |
| Hypnotic drugs | 0.452 | 0.294 |
| Psychotropic drugs | 1.045 | 0.786 |
| Age group | 0.298 | 0.24 |
| Adl_standing | Chi-Square | 9.05E-23 |
| Adl_standing | Chi-Square | 9.05E-23 |
| Adl_standing | Chi-Square | 9.05E-23 |
| Adl_standing | Chi-Square | 9.05E-23 |
| Mmtright | 0.0 | 0.0 |
| Mmtleft | 0.0 | 0.0 |

**Table S5b. Hyperparameters for machine learning models**

| Model | Hyperparameters Tuned | Tuning Method | Best Parameters |
| --- | --- | --- | --- |
| RF | n_estimators (100), max_depth (10), min_samples_split (2), criterion (gini) | Grid Search with Cross-validation | n_estimators=100, max_depth=10, criterion=gini |
| LR | C (1.0), penalty (l2), max_iter (100) | Grid Search with Cross-validation | C=1.0, penalty=l2 |
| XGB | n_estimators (100), learning_rate (0.1), max_depth (6) | Grid Search with Cross-validation | n_estimators=100, learning_rate=0.1, max_depth=6 |
| GBDT | n_estimators (100), learning_rate (0.1), max_depth (3) | Grid Search with Cross-validation | n_estimators=100, learning_rate=0.1, max_depth=3 |
| GNB | No hyperparameter tuning required (assumes default values) | Not applicable | Default parameters |
| MLP | hidden_layer_sizes (100), activation (relu), solver (adam) | Random Search with Cross-validation | hidden_layer_sizes=100, activation=relu |
| SVM | C (1.0), kernel (rbf), gamma (scale) | Grid Search with Cross-validation | C=1.0, kernel=rbf, gamma=scale |
| KNN | n_neighbors (5), weights (uniform), algorithm (auto) | Grid Search with Cross-validation | n_neighbors=5, weights=uniform |

**Table S6-a. Multi-model classification - Summary of training set results.**

| **Classification model** | **AUC (95% CI)** | **Cutoff (95% CI)** | **Accuracy (95% CI)** | **Sensitivity (95% CI)** | **Specificity (95% CI)** | **Positive predictive value (95% CI)** | **Negative predictive value (95% CI)** | **F1-score (95% CI)** | **Kappa (95% CI)** |
| --- | --- | --- | --- | --- | --- | --- | --- | --- | --- |
| XGBoost | 0.869 (0.857-0.881) | 0.509(0.490-0.528) | 0.784(0.781-0.787) | 0.754(0.727-0.780) | 0.814(0.792-0.836) | 0.803(0.789-0.817) | 0.768(0.754-0.782) | 0.777(0.769-0.785) | 0.568(0.561-0.574) |
| LR | 0.767 (0.751-0.784) | 0.529(0.464-0.595) | 0.709(0.704-0.714) | 0.647(0.586-0.709) | 0.771(0.707-0.834) | 0.744(0.709-0.778) | 0.689(0.668-0.709) | 0.689(0.669-0.709) | 0.418(0.408-0.428) |
| RF | 0.871 (0.859-0.883) | 0.508(0.481-0.535) | 0.784(0.782-0.787) | 0.74(0.716-0.763) | 0.829(0.808-0.850) | 0.813(0.799-0.827) | 0.761(0.750-0.773) | 0.774(0.767-0.781) | 0.569(0.563-0.574) |
| GBDT | 0.810 (0.795-0.825) | 0.515(0.491-0.538) | 0.741(0.735-0.746) | 0.699(0.679-0.718) | 0.783(0.768-0.797) | 0.763(0.755-0.771) | 0.722(0.712-0.732) | 0.729(0.720-0.738) | 0.481(0.470-0.492) |
| GNB | 0.762 (0.746-0.779) | 0.54(0.496-0.583) | 0.713(0.708-0.719) | 0.645(0.627-0.664) | 0.782(0.773-0.791) | 0.747(0.743-0.752) | 0.688(0.679-0.697) | 0.692(0.682-0.703) | 0.427(0.415-0.439) |
| MLP | 0.774 (0.758-0.790) | 0.539(0.501-0.577) | 0.715(0.707-0.723) | 0.659(0.628-0.689) | 0.771(0.737-0.804) | 0.743(0.723-0.763) | 0.694(0.682-0.705) | 0.697(0.686-0.709) | 0.429(0.413-0.446) |
| SVM | 0.777 (0.761-0.794) | 0.677(0.654-0.700) | 0.731(0.726-0.736) | 0.634(0.617-0.652) | 0.827(0.813-0.840) | 0.786(0.776-0.796) | 0.694(0.686-0.701) | 0.702(0.693-0.711) | 0.461(0.451-0.471) |
| KNN | 0.713 (0.699-0.726) | 0.2(0.200-0.200) | 0.691(0.688-0.694) | 0.428(0.421-0.434) | 0.954(0.951-0.958) | 0.903(0.897-0.909) | 0.625(0.622-0.628) | 0.58(0.574-0.586) | 0.382(0.376-0.388) |

**Table S6-b. Multi-model classification-validation set result summary.**

| **Classification model** | **AUC (95% CI)** | **Cutoff (95% CI)** | **Accuracy (95% CI)** | **Sensitivity (95% CI)** | **Specificity (95% CI)** | **Positive predictive value (95% CI)** | **Negative predictive value (95% CI)** | **F1-score (95% CI)** | **Kappa (95% CI)** |
| --- | --- | --- | --- | --- | --- | --- | --- | --- | --- |
| XGBoost | 0.847 (0.820-0.873) | 0.509(0.490-0.528) | 0.766(0.749-0.782) | 0.735(0.700-0.770) | 0.796(0.769-0.824) | 0.784(0.764-0.803) | 0.751(0.729-0.774) | 0.758(0.738-0.778) | 0.532(0.499-0.564) |
| LR | 0.765 (0.732-0.798) | 0.529(0.464-0.595) | 0.702(0.680-0.724) | 0.637(0.549-0.726) | 0.766(0.689-0.844) | 0.739(0.692-0.786) | 0.684(0.645-0.723) | 0.679(0.640-0.717) | 0.404(0.361-0.447) |
| RF | 0.850 (0.824-0.876) | 0.508(0.481-0.535) | 0.769(0.752-0.785) | 0.725(0.689-0.761) | 0.812(0.780-0.844) | 0.795(0.770-0.821) | 0.748(0.726-0.770) | 0.758(0.738-0.777) | 0.537(0.503-0.571) |
| GBDT | 0.797 (0.766-0.828) | 0.515(0.491-0.538) | 0.732(0.711-0.754) | 0.695(0.675-0.714) | 0.77(0.740-0.801) | 0.752(0.724-0.780) | 0.716(0.697-0.735) | 0.722(0.701-0.743) | 0.465(0.421-0.509) |
| GNB | 0.762 (0.729-0.795) | 0.54(0.496-0.583) | 0.707(0.679-0.735) | 0.64(0.603-0.678) | 0.773(0.734-0.813) | 0.74(0.704-0.775) | 0.683(0.658-0.708) | 0.686(0.656-0.716) | 0.414(0.359-0.469) |
| MLP | 0.771 (0.738-0.803) | 0.539(0.501-0.577) | 0.708(0.684-0.731) | 0.649(0.587-0.712) | 0.766(0.718-0.814) | 0.737(0.705-0.769) | 0.688(0.656-0.721) | 0.688(0.655-0.722) | 0.416(0.369-0.462) |
| SVM | 0.765 (0.732-0.798) | 0.677(0.654-0.700) | 0.725(0.704-0.745) | 0.628(0.599-0.657) | 0.822(0.807-0.836) | 0.778(0.758-0.799) | 0.689(0.669-0.708) | 0.695(0.670-0.720) | 0.45(0.409-0.490) |
| KNN | 0.703 (0.675-0.731) | 0.2(0.200-0.200) | 0.682(0.663-0.701) | 0.425(0.384-0.466) | 0.939(0.929-0.949) | 0.874(0.859-0.890) | 0.621(0.604-0.637) | 0.571(0.532-0.609) | 0.364(0.325-0.403) |

**Table S6-c. Summary of Mean z-values for DeLong's Test**

| Name | XGB | LR | RF | GBDT | GNB | MLP | SVC | KNN |
| --- | --- | --- | --- | --- | --- | --- | --- | --- |
| XGB | NA | 6.865 | 1.278 | 5.69 | 6.899 | 6.547 | 7.027 | 11.136 |
| LR | 6.865 | NA | 7.14 | 4.534 | 1.137 | 1.72 | 0.964 | 4.23 |
| RF | 1.278 | 7.14 | NA | 5.911 | 7.214 | 6.816 | 7.271 | 11.603 |
| GBDT | 5.69 | 4.534 | 5.911 | NA | 4.267 | 3.711 | 3.938 | 6.679 |
| GNB | 6.899 | 1.137 | 7.214 | 4.267 | NA | 1.953 | 0.662 | 4.036 |
| MLP | 6.547 | 1.72 | 6.816 | 3.711 | 1.953 | NA | 1.345 | 4.656 |
| SVC | 7.027 | 0.964 | 7.271 | 3.938 | 0.662 | 1.345 | NA | 3.984 |
| KNN | 11.136 | 4.23 | 11.603 | 6.679 | 4.036 | 4.656 | 3.984 | NA |

**Table S6-d. Summary of Mean p-values from DeLong’s Test**

| Name | XGB | LR | RF | GBDT | GNB | MLP | SVC | KNN |
| --- | --- | --- | --- | --- | --- | --- | --- | --- |
| XGB | NA | 0.0 | 0.321 | 0.0 | 0.0 | 0.0 | 0.0 | 0.0 |
| LR | 0.0 | NA | 0.0 | 0.0 | 0.509 | 0.174 | 0.475 | 0.001 |
| RF | 0.321 | 0.0 | NA | 0.0 | 0.0 | 0.0 | 0.0 | 0.0 |
| GBDT | 0.0 | 0.0 | 0.0 | NA | 0.0 | 0.01 | 0.004 | 0.0 |
| GNB | 0.0 | 0.509 | 0.0 | 0.0 | NA | 0.168 | 0.601 | 0.003 |
| MLP | 0.0 | 0.174 | 0.0 | 0.01 | 0.168 | NA | 0.273 | 0.0 |
| SVC | 0.0 | 0.475 | 0.0 | 0.004 | 0.601 | 0.273 | NA | 0.003 |
| KNN | 0.0 | 0.001 | 0.0 | 0.0 | 0.003 | 0.0 | 0.003 | NA |

**TABLE S7. Summary of Results for the Training, Validation, and Test Sets Using the Random Forest Machine Learning Approach**

|  | AUC (95% CI) | Cutoff (95% CI) | Accuracy (95% CI) | Sensitivity (95% CI) | Specificity (95% CI) | Positive predictive value (95% CI) | Negative predictive value (95% CI) | F1-score (95% CI) |
| --- | --- | --- | --- | --- | --- | --- | --- | --- |
| Training | 0.814(0.802-0.827) | 0.518(0.509-0.528) | 0.720(0.719-0.722) | 0.685(0.669-0.702) | 0.757(0.742-0.772) | 0.747(0.740-0.755) | 0.697(0.691-0.704) | 0.714(0.709-0.720) |
| Validation | 0.781(0.740-0.822) | 0.518(0.509-0.528) | 0.691(0.678-0.703) | 0.659(0.638-0.681) | 0.723(0.696-0.750) | 0.715(0.697-0.732) | 0.670(0.658-0.683) | 0.685(0.672-0.698) |
| Test | 0.794 (0.769-0.819) | 0.52 | 0.714 | 0.719 | 0.708 | 0.719 | 0.708 | 0.719 |

**Table S8. Meaning and interpretation of various performance metrics in machine learning classification tasks.**

| **Metric** | **Significance** | **Interpretation** |
| --- | --- | --- |
| **AUC** | Measures the model's ability to discriminate between classes | AUC ranges from 0.5 (random) to 1 (perfect discrimination). Higher AUC indicates better overall performance. |
| **Cutoff** | Threshold value for classification | Determines the point at which predicted probabilities are classified as positive or negative. |
| **Accuracy** | Proportion of correctly classified instances | Accuracy = (TP + TN) / (TP + TN + FP + FN). High accuracy indicates a higher proportion of correct predictions. |
| **Sensitivity** (Recall) | Proportion of true positives correctly classified | Sensitivity = TP / (TP + FN). Measures how well the model identifies positive instances. |
| **Specificity** | Proportion of true negatives correctly classified | Specificity = TN / (TN + FP). Measures how well the model identifies negative instances. |
| **Positive Predictive Value** (Precision) | Proportion of true positives among positive predictions | PPV = TP / (TP + FP). Measures the accuracy of positive predictions. |
| **Negative Predictive Value** | Proportion of true negatives among negative predictions | NPV = TN / (TN + FN). Measures the accuracy of negative predictions. |
| **F1-**Score | Harmonic mean of precision and recall | F1 Score = 2 * (Precision * Recall) / (Precision + Recall). Balances precision and recall for imbalanced datasets. |
| **Kappa** | Measures the agreement between predicted and actual labels | Kappa measures inter-rater agreement, with 1 indicating perfect agreement and 0 indicating random agreement. |

AUC (Area Under the ROC Curve); CI (Confidence Interval); FN (False Negative); FP (False Positive); NPV (Negative Predictive Value); PPV (Positive Predictive Value); ROC (Receiver Operator Characteristic); TN (True Negative); TP (True Positive)

**Supplementary Figures**


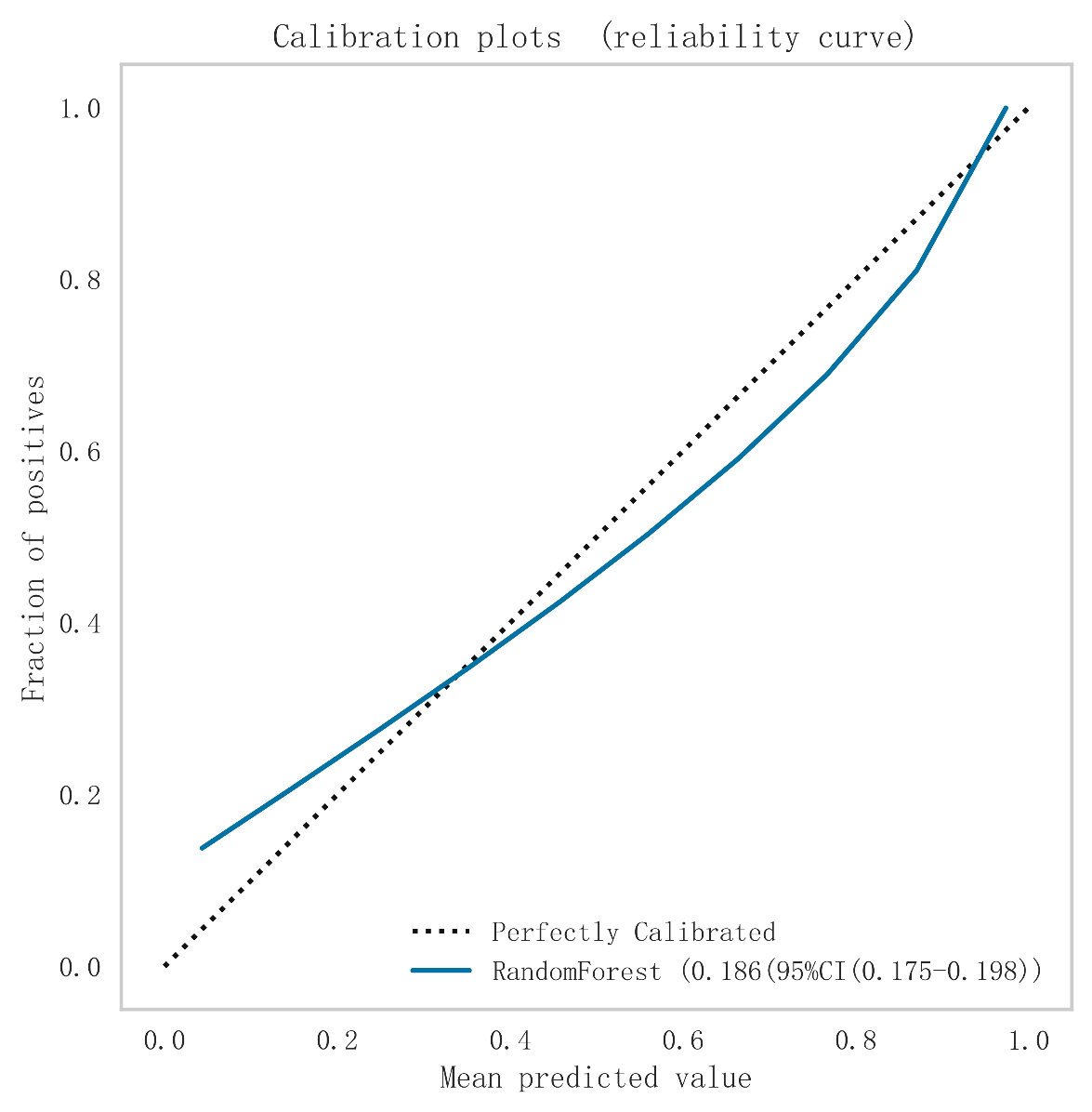


**Figure S1a.** Calibration plot for the Random Forest model. The solid blue line represents the model's predicted probabilities compared to actual outcomes, while the dashed line indicates perfect calibration. The closer the blue line is to the dashed line, the better the model is calibrated. The model's AUC is 0.186 (95% CI: 0.175–0.198).


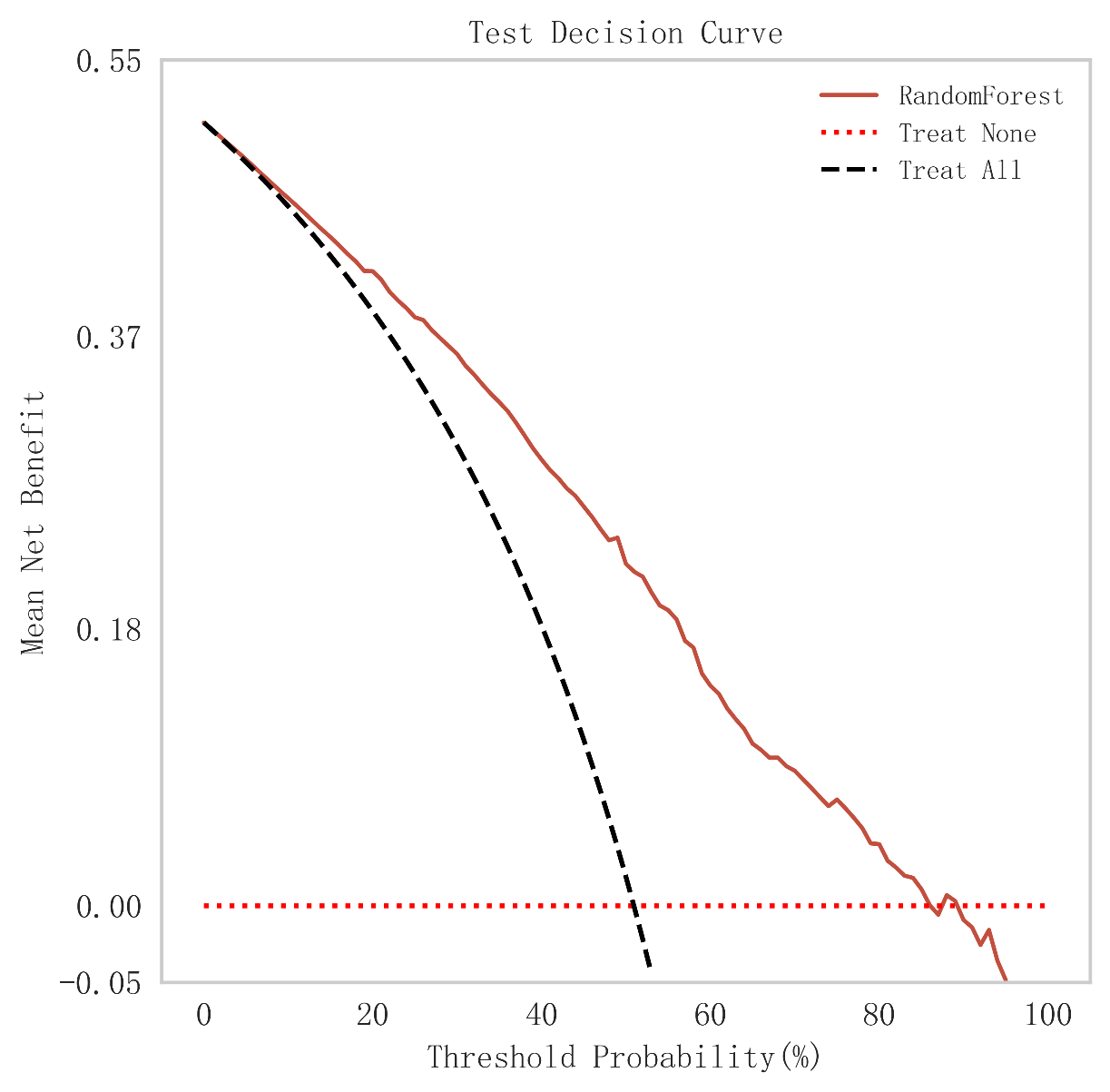


**Figure S1b.** Decision curve analysis for the Random Forest model. The solid red line represents the net benefit of the Random Forest model across different threshold probabilities. The dashed black line indicates the net benefit of treating all patients, while the dotted red line represents treating none. The Random Forest model provides higher net benefit compared to treating all or none across a wide range of threshold probabilities.
